# Parma consensus statement on metabolic disruptors

**DOI:** 10.1186/s12940-015-0042-7

**Published:** 2015-06-20

**Authors:** Jerrold J. Heindel, Frederick S. vom Saal, Bruce Blumberg, Patrizia Bovolin, Gemma Calamandrei, Graziano Ceresini, Barbara A. Cohn, Elena Fabbri, Laura Gioiosa, Christopher Kassotis, Juliette Legler, Michele La Merrill, Laura Rizzir, Ronit Machtinger, Alberto Mantovani, Michelle A. Mendez, Luisa Montanini, Laura Molteni, Susan C. Nagel, Stefano Parmigiani, Giancarlo Panzica, Silvia Paterlini, Valentina Pomatto, Jérôme Ruzzin, Giorgio Sartor, Thaddeus T. Schug, Maria E. Street, Alexander Suvorov, Riccardo Volpi, R. Thomas Zoeller, Paola Palanza

**Affiliations:** Division of Extramural Research and Training, National Institute of Environmental Health Sciences, Research Triangle Park, NC USA; Division of Biological Sciences, University of Missouri, Columbia, MO USA; Department of Developmental and Cell Biology, University of California, Irvine, CA USA; Department of Life Sciences and Systems Biology, University of Turin, Turin, Italy; Department of Cell Biology and Neurosciences, Insituto Superiore di Sanita, Rome, Italy; Department of Clinical and Experimental Medicine, University of Parma, Parma, Italy; Public Health Institute, Berkeley, CA USA; Interdepartment Center for Environmental Science Research, University of Bologna, Ravenna, Italy; Department of Neuroscience, University of Parma, Parma, Italy; Department of Toxicology and Environmental Health, VU University Amsterdam, Amsterdam, Netherlands; Department of Environmental Toxicology, University of California, Davis, CA USA; Department of Health Sciences, University of Milano-Bicocca, Monza, Italy; Sheba Medical Center and Tel-Aviv University, Tel –Aviv, Israel; Instituto Superiore di Sanita, Rome, Italy; School of Public Health, University of North Carolina at Chapel Hill, Chapel Hill, NC USA; Department of Pediatrics, University of Parma, Parma, Italy; University of Milano-Bicocca, Monza, Italy; Department of Obstetrics, Gynecology and Women’s Health, University of Missouri, Columbia, MO USA; Faculty of Medicine, University of Parma, Parma, Italy; Department of Neuroscience and Neuroscience Institute Cavalieri Ottolenghi (NICO), University of Turin, Turin, Italy; Department of Biology, University of Bergen, Bergen, Norway; Department of Pharmacy and Biotechnology, University of Bologna, Bologna, Italy; Department of Pediatrics, University Hospital, Parma, Italy; Department of Internal Medicine, University of Parma, Parma, Italy; Department of Biology, University of Massachusetts, Amherst, MA USA

**Keywords:** Metabolic disruptor, Obesogen, Obesity, Diabetes, Metabolic syndrome, Developmental Programming

## Abstract

A multidisciplinary group of experts gathered in Parma Italy for a workshop hosted by the University of Parma, May 16–18, 2014 to address concerns about the potential relationship between environmental metabolic disrupting chemicals, obesity and related metabolic disorders. The objectives of the workshop were to: 1. Review findings related to the role of environmental chemicals, referred to as “metabolic disruptors”, in obesity and metabolic syndrome with special attention to recent discoveries from animal model and epidemiology studies; 2. Identify conclusions that could be drawn with confidence from existing animal and human data; 3. Develop predictions based on current data; and 4. Identify critical knowledge gaps and areas of uncertainty. The consensus statements are intended to aid in expanding understanding of the role of metabolic disruptors in the obesity and metabolic disease epidemics, to move the field forward by assessing the current state of the science and to identify research needs on the role of environmental chemical exposures in these diseases. We propose broadening the definition of obesogens to that of metabolic disruptors, to encompass chemicals that play a role in altered susceptibility to obesity, diabetes and related metabolic disorders including metabolic syndrome.

## Introduction

### Obesity and related metabolic diseases

There is now convincing evidence that shows a dramatic increase in the incidence of a set of related clinical phenotypes and diseases linked to the metabolic syndrome over the last 4 decades. Metabolic syndrome is characterized by a cluster of symptoms including insulin resistance, hyperglycemia, abdominal obesity, dyslipidemia, and hypertension [1] that occur together, increasing risk for type 2 diabetes, cardiovascular disease (angina, hypertension, stroke, heart attack), liver diseases (steatosis), inflammatory and immune disorders [1, 2]. Obesity is defined as having a body mass index (BMI) > 30 kg/square meter and has increased from 13 % to 35 % in adults over the last 4 decades in the United States [3]. Type 2 diabetes increased by 30.5 % in children and adolescents in the USA between 2001–2009 [4]. Type 2 was once referred to as “adult onset diabetes”, but it is now rapidly increasing in incidence in children and adolescents concurrently with the increase in childhood obesity [5]. Visceral (intra-abdominal) fat accumulation appears to be a major risk factor for the other components of metabolic syndrome [6]. Finally, the increasing incidence of metabolic syndrome is recognized as a global health problem, not just a problem of developed countries [7].

The current focus on the etiology of increased obesity remains on imbalance between food intake and energy expenditure. Although clinicians continue to recommend reducing calorie intake and increasing exercise [6], data show that once a person is overweight or obese, effective long term treatment is difficult. More than 90 % of those who achieve significant weight loss do not maintain it. Obesity is a complex endocrine disease caused by disruption of many hormonal control systems that link the gastro-intestinal tract, pancreas, muscle, adipose tissue, liver and brain, and involves an interaction between genetic and environmental factors. Furthermore the hypothalamic pituitary axis and or and/or hypothalamic gonadal axis function affects in turn energy balance and metabolic function.

Given the limited success reversing the obesity epidemic and related morbidities by focusing solely on nutrition, exercise or drug therapies, there is a compelling need to consider other potential environmental factors that could be major contributors to visceral adiposity and other aspects of metabolic syndrome that are increasing at a dramatic rate worldwide. For example, a recent study showed that animals (feral animals in cities, pets, laboratory animals) living in proximity to humans are becoming obese in parallel with humans over the past few decades [8]. This observation strongly supports the existence of additional factors beyond energy balance in the obesity epidemic.

### Endocrine disruption and the Developmental Origins of Health and Disease (DOHaD)

Extensive evidence from human and animal research shows that disruption of metabolic systems during “critical windows” in development can lead to permanent changes that impact the likelihood of developing a variety of non-communicable diseases. These include obesity and components of metabolic syndrome, with the specific systems affected determined by the type and timing of developmental exposure. This Developmental Origins of Health and Disease (DOHaD) hypothesis [2, 9] holds that early life (e.g., in utero development, the neonatal period and childhood, when tissues and body systems are developing and malleable) is a extraordinarily sensitive time during which stressors (e.g., diet or EDCs) can alter gene expression, protein levels, cell numbers, fate or locations to cause changes in tissue and organ function. These changes persist after the stressor is gone and lead to increased susceptibility to disease and dysfunctions later in life. There are no known classical genetic mechanisms that could explain the remarkable changes in body composition that have occurred over recent decades. Therefore, there has been a significant focus on identifying changes in gene expression and epigenetic marks caused by environmental factors, such as stress [10], drugs (for example nicotine) and a number of endocrine disrupting chemicals during development (in utero and early childhood) in relation to the risk of metabolic diseases later in life [11–14].

### The obesogen hypothesis

Endocrine disrupting chemicals (EDCs) are a subclass of toxic chemicals that act by altering some aspect of hormone action (reviewed in [15]). Animal and human exposure data show that a variety of EDCs can act as “obesogens”, during development with exposures during critical periods in organogenesis, around puberty and in adulthood being related to an increase in central fat deposition and function [16–19]. Obesogens can act to promote obesity by increasing the number and size of fat cells, by shifting energy balance to favor calorie storage, by altering basal metabolic rate, by altering gut microbiota to promote food storage [20] by altering hormonal control of appetite and satiety [17, 18, 21, 22] and by altering brain circuitries controlling food intake and energy expenditure [23]. Some EDCs have been found to impact insulin levels together with vascular, immune, liver and cardiovascular function.

New obesogenic chemicals are being identified at an increasing rate. These include estrogenic EDCs such as diethylstilbestrol (DES) [24], bisphenol A (BPA) [25, 26], DDT [27, 28], organotins such as tributyltin (TBT) [29, 30], perfluorooctanoates [31] and phthalates [32–34]. All of these chemicals show obesogenic properties in laboratory animals. In humans urinary phthalate levels have been correlated with increased waist diameter [35, 36] and urinary BPA has been correlated with obesity in children and adolescents [37, 38] and adults [39]. Several persistent organic pollutants (e.g., DDE, HCB, polybrominated diphenyl ethers) have been linked with obesity in humans [40, 41].

The effects of prenatal exposure to some obesogens persist until at least the F3 generation. This is significant because in multigeneration studies, F0 and F1 animals are directly exposed to the test chemical; F2 are exposed as germ cells within F1 animals. The F3 is the first generation that has not received any direct chemical exposure; therefore, effects observed in F3 and beyond are considered to be transgenerational and permanent. Transgenerational effects are distinct from the multigenerational effects in F1 and F2 animals [14, 42] ( see reference 41 for figure depicting transgenerational effects). Exposure of pregnant F0 animals to low, environmentally-relevant levels of TBT in their drinking water led to increased fat depot size, reprogramming of multipotent mesenchymal stem cells toward the adipogenic lineage and hepatic steatosis through the F3 generation [29]. Prenatal TBT exposure is thus a maternal programming event that permanently altered stem cell fate leading to a reproducible adult phenotype. Skinner and colleagues have shown that plastic components such as BPA, diethylhexyl phthalate and dibutyl phthalate [34], a mixed hydrocarbon mixture (jet fuel JP-8) [43], and the once widely used environmentally persistent pesticide, DDT [27], all lead to a transgenerational predisposition to obesity in the F3 generation (among other adverse outcomes). Taken together, these observations support the relevance of the obesogen hypothesis. Nonetheless the obesogen hypothesis has been largely overlooked by the medical community as a contributing factor to obesity.

#### Parma meeting

A multidisciplinary group of experts (epidemiologists, biologists, toxicologists, endocrinologists, neurobiologists, risk assessors, molecular biologists, and clinicians with an interest in the role of environmental chemicals in obesity, diabetes and metabolic syndrome) gathered in Parma Italy for a workshop/brainstorming session hosted by the University of Parma, May 16–18, 2014. This was the first international working group to focus on the role of environmental chemicals exposures in obesity and metabolic syndrome. The objective of the conference was to bring together scientists from a variety of disciplines to examine the relationship between EDC exposure and the increased incidence of metabolic disorders in many populations, including obesity, diabetes, cardiovascular disease, hypertension and kidney disease (Table 1).

**Table 1:** The objectives of the Parma workshop were to

• Review findings related to the role of environmental chemicals in obesity and metabolic syndrome with special attention to recent discoveries from animal model and epidemiology studies.
• Discuss refocusing the science from obesogens (obesity focus) to a more general focus on metabolic disruptors (metabolic syndrome).
• Identify conclusions that could be drawn with confidence from existing animal and human data.
• Develop predictions based on current data.
• Identify critical knowledge gaps and areas of uncertainty.
• Develop ideas and plans for future research that would help fill data gaps and resolve uncertainties that would lead to greater understanding of the obesogen/metabolic disruptor paradigm by the wider scientific and general communities.
• Develop plans to stimulate outreach to a wider scientific community via symposia, review articles, teleconferences.

The workshop opened with a general discussion of the state of the science linking endocrine disruptors with obesity/diabetes/metabolic syndrome. The following day there were sessions discussing evidence from animal models, human epidemiology studies, integrating human and animal data, expanding endpoints in animal and human studies, improving study designs, windows of susceptibility and developmental programming and epigenetics. The final session focused on defining a path forward, including integration of the obesogen/metabolic disruptor hypothesis into mainstream science. The results of this workshop are presented here as a series of consensus statements indicating what is known, where there are data gaps, and what is needed to move this field forward to prevent obesity, diabetes and other co-morbidities related to metabolic disruption.

A major conclusion from the workshop was that the word, obesogen, while it served an important purpose to focus interest on EDCs and obesity, had become too restrictive. While there may be chemicals that only increase susceptibility to obesity (and are appropriately called obesogens), there are environmental chemicals that can cause other aspects of metabolic syndrome as well as diabetes. For example, humans are exposed to bisphenol A via polycarbonate plastics, many can linings and some cash register receipts; bisphenol A exposure in rodent models results in increased weight, altered glucose homeostasis, altered beta cell function, altered liver lipids and cardiovascular dysfunction [26, 44–46]. How many other chemicals would have multiple effects on metabolism remains an open question. Thus, it was decided that the field should change from using the specific word obesogen when referring to diseases other than obesity to the more general term, “metabolic disruptor” as originally proposed by Casas-Casas and Desvergne [47].

#### “Metabolic disruptor” hypothesis

We hypothesize that environmental chemicals can act during development and/or other sensitive time periods across the lifespan to control adipose tissue development by increasing the number and/or size of fat cells and/or by altering food intake and metabolism via specific effects on the brain, pancreas, adipose tissue, liver, GI tract and muscle individually or in combination. These metabolic disruptors thereby alter programming or sensitivity for developing obesity/diabetes or aspects of metabolic syndrome later in life.

Increased susceptibility to obesity/diabetes/metabolic syndrome may result directly from exposure to the metabolic disruptor or in other cases may require a second “hit”, for example, increased fat or sugar in the diet and/or stress for the functional change to be expressed as a phenotype. The effects may be sexually dimorphic and the dose responses may be non-monotonic in nature as is often the case for EDCs [48, 49].

#### Consensus statement

We are confident of the following:

- There is a global increase in incidence of obesity, diabetes and metabolic diseases.
- There is a global increase in childhood obesity and type 2 diabetes.
- While there are genes that play important roles in these diseases, like all complex diseases there must be both genetic and environmental components.
- The increase in obesity and metabolic diseases over the last 4 decades cannot be accounted for by classical genetic factors, and thus must be due to some aspect of the environment.
- There is more to the environmental component of obesity, diabetes and metabolic syndrome than overeating and poor nutrition, lack of exercise and changes in lifestyle. The environmental component is multifactorial and includes prescription drugs, stress, nutrition, microbiome, infections, sleep patterns, nocturnal illumination and environmental chemicals.
- Obesity and metabolic syndrome are endocrine diseases/dysfunctions and thus sensitive to disruption by environmental agents that can interfere with hormone and neuroendocrine action (e.g. EDCs).
- There are pharmaceutical obesogens - prescription drugs with the known side effect of causing weight gain.
- Susceptibility to metabolic disorders is, at least in part, ‘programmed’ *in utero* and early postnatal life by exposure to environmental factors including stress, drugs, nutrition *and* en*v*ironmental chemicals.
- Programming may alter brain appetite and/or satiety centers as well as fat cell numbers and other aspects of metabolism, including effects on control of the GI tract, muscle, pancreas, liver functions, etc. (e.g. altering the sensitivity for gaining weight).

We estimate with confidence that:

- Effects will be due to “multiple hits” of environmental exposures and may occur only after a latent period of months to decades, requiring a lifespan research approach, including prospective human studies.
- There are multiple specific windows of enhanced susceptibility to metabolic disruptors across the lifespan, including paternal, *in utero*, early childhood, pre-puberty, pregnancy (for the mother) menopause and aging.
- Development, in *utero* and during the first few years of life, is the most sensitive window of susceptibility for metabolic disruption.
- The two sexes show differential susceptibility to metabolic disruption as well as different critical windows for, and different effects of, exposure.
- Understanding environmental effects on these diseases requires sensitive measures of personal exposures and sensitive endpoints to identify phenotypes.
- Effects of EDC exposure will vary depending on co-occurrence of other environmental stressors such as prescription drugs, sleep, hypercaloric diet, activity, stress, socioeconomic status, infections, microbiome, anxiety-depression etc., requiring a detailed analysis of potential interacting and confounding factors.

Existing data lead us to predict that:

- The effects of metabolic disruptors may be difficult to detect at the individual level due to human genomic variability creating a heterogeneous population requiring a genomic and statistical approach.
- Some effects of metabolic disruptors may be transgenerational, requiring a multigenerational approach: a minimum of two generations for paternal line effects and three generations for maternal line effects.
- Effects of metabolic disruptors will likely be dependent on the dose and route of exposure and may exhibit non-monotonic dose responses; this will require dose response studies and a pharmacokinetic approach.
- We should expect effects to be due to multiple chemicals with varying half-lives, metabolism, persistence, tissue accumulation and target sensitivities; complete analysis will require a mixtures approach.
- Certain metabolic disruptors will have specific actions, causing only obesity, diabetes or altered liver function whereas others will affect many aspects of metabolism leading to metabolic syndrome.
- We are underestimating the importance of metabolic disruptors in obesity, diabetes, and metabolic syndrome because current research designs focus on studying one or a small subset of chemicals at a time, during limited windows of sensitivity, in single tissues (including only one adipose tissue) and often only endpoints related to a single disease outcome per study.
- Reducing exposures to environmental chemicals and improving nutrition during development offers the possibility of preventing obesity and metabolic diseases.
- The totality of environmental effects on obesity (drugs, chemicals, stress and nutrition) will likely be greater than the effects of genetic predisposition.

#### Focus of future research

We believe a multidisciplinary and integrated research strategy is needed to further test the hypothesis that metabolic disruptors alter the sensitivity to develop obesity, diabetes and metabolic syndrome. To obtain meaningful results in a reasonable time and improve health and well-being of future generations, we believe that future research should focus on:

- Characterizing adverse outcome pathways through which metabolic disruptors lead to different manifestations of metabolic syndrome.
- Identifying windows of susceptibility, how many are there, what the mechanisms are underlying a window of sensitivity and how exposures to metabolic disruptors in multiple windows (e.g. developmental and later life exposures) interact across the lifespan and generations.
- Defining the role of metabolic disruptors in type 1 and 2 diabetes.
- Examining multiple endpoints to determine whether a chemical leads to multiple metabolic disorders or only one or a subset of metabolic diseases. Appropriate endpoints include adipose tissue depots (including brown adipose tissue), insulin and glucose metabolism, liver function focusing on lipid metabolism, muscle metabolism, inflammation, feeding behavior and neural networks controlling food intake, food preference and satiety patterns, GI effects and measures of hypertension and cardiovascular disease, and the interaction between the gut microbiome and EDC exposure.
- Assessing epigenomic and other markers underlying altered developmental programming of metabolic functions and endpoints in human studies and in animal models.
- Addressing susceptible exposure windows and multiple outcome windows over the life course that use mother-child cohorts and bio-banks.
- Developing studies to determine whether metabolic disruptors alter the “set-point” or sensitivity for gaining weight and the ability to lose weight and the mechanism(s) for these effects.
- Examining sex differences and differences in adipose depots in responses.
- Developing early biomarkers from developmental exposure to environmental agents that are associated with underlying causal mechanisms that can predict disease outcomes later in life.
- Developing and validating in *vivo* and *in vitro* screens to detect and prioritize metabolic disruptors.
- Adding robust and relevant endpoints to guideline studies (used by regulatory agencies) to detect various aspects of metabolic disruption other than just body weight.
- Adding endpoints related to metabolism and metabolic rate to assess energy efficiency.
- Improving exposure assessments in human studies: integrate genetics, genomics, proteomics and metabolomics with environmental exposures to better understand the role of metabolic disruptors in disease onset and severity.
- Assessing multiple chemicals, mixture studies, and integration with other stressors including stress, drugs, nutrition and infections.
- Developing an integrated conceptual approach linking animal studies and endpoints with longitudinal human cohort studies; improved collaborations among epidemiologists, clinicians and animal researchers through integrated research projects, comparable markers of exposure assessment and effects. Integrated scientific meetings will greatly aid in the integration of these scientific fields with that of metabolic disruptor research.

## Summary and conclusions

The Parma workshop helped to focus this emerging field by developing an overarching hypothesis for the role of environmental chemicals in the current worldwide epidemics of obesity, diabetes and related metabolic diseases. We hope that the consensus statements will aid in expanding understanding of the possible role of metabolic disruptors in these epidemics and have identified research needs in order to provide more relevant data on the role of environmental chemicals in these diseases. The objective is both to indicate the strength of the current data and to provide a roadmap for further studies. A coherent, enhanced research agenda will help identify strategies to prevent metabolic diseases through actions that can be taken by individuals as well as public health agencies. History shows that prevention is always the best strategy. Increased understanding of the importance of the metabolic disruptor hypothesis to the epidemics of obesity and metabolic syndrome offers the potential for these diseases to be mitigated by modifying exposures, thereby creating a healthier environment for future generations.

## Abbreviations

BPA: Bisphenol A
BMI: Body mass index
EDCs: Endocrine disrupting chemicals
DDE: Dichlorodiphenyldichloroethylene
DDT: Dichlorodiphenyltrichloroethane
DES: Diethylstilbestrol
GI: Gastro-intestinal
HCB: Hexachlorobenzene
TBT: Tributyltin
HPA: Hypothalamic pituitary adrenal
HPG: Hypothalamic pituitary gonadal

## References

[1] O’Neill S, O’Driscoll L (2015). Metabolic syndrome: a closer look at the growing epidemic and its associated pathologies. Obes Rev.

[2] Heindel JJ, vom Saal FS (2009). Role of nutrition and environmental endocrine disrupting chemicals during the perinatal period on the aetiology of obesity. Mol Cell Endocrinol.

[3] Ogden CL, Carroll MD, Flegal KM (2014). Prevalence of obesity in the United States. JAMA.

[4] Burke JP, Williams K, Gaskill SP, Hazuda HP, Haffner SM, Stern MP (1999). Rapid rise in the incidence of type 2 diabetes from 1987 to 1996: results from the San Antonio Heart Study. Arch Intern Med.

[5] Dabelea D, Mayer-Davis EJ, Saydah S, Imperatore G, Linder B, Divers J, Bell R, Badaru A, Talton JW, Crume T (2014). Prevalence of type 1 and type 2 diabetes among children and adolescents from 2001 to 2009. JAMA.

[6] Despres JP (2012). Abdominal obesity and cardiovascular disease: is inflammation the missing link?. Canadian J Cardiol.

[7] Amuna P, Zotor FB (2008). Epidemiological and nutrition transition in developing countries: impact on human health and development. Proc Nutrit Socy.

[8] Klimentidis YC (2011). Canaries in the coal mine: a cross-species analysis of plurality of obesity epidemics. Proc Biol Sci.

[9] Hanson MA, Gluckman PD (2011). Developmental origins of health and disease: Moving from biological concepts to interventions and policy. Int J Gynecol Obstetrics.

[10] Anacker C, O’Donnell KJ, Meaney MJ (2014). Early life adversity and the epigenetic programming of hypothalamic-pituitary-adrenal function. Dialogues Clin Neurosci.

[11] La Merrill MA, Cirillo PM, Krigbaum N, Cohn BA: The impact of prenatal parental tobacco smoking on risk of diabetes mellitus in middle-aged women in review. J dev orig health res. 2015;6(3):242–9. 10.1017/S2040174415000045 PMC6996969 25665487

[12] La Merrill M, Cirillo PM, Terry MB, Krigbaum NY, Flom JD, Cohn BA (2013). Prenatal Exposure to the Pesticide DDT and Hypertension Diagnosed in Women before Age 50: A Longitudinal Birth Cohort Study. Environ Health Perspect.

[13] Skinner MK (2014). Endocrine disruptor induction of epigenetic transgenerational inheritance of disease. Mol Cell Endocrinol.

[14] McCarrey JR (2014). Distinctions between transgenerational and non-transgenerational epimutations. Mol Cell Endocrinol.

[15] Zoeller RT, Bergman A, Becher G, Bjerregaard P, Bornman R, Brandt I, Iguchi T, Jobling S, Kidd KA, Kortenkamp A (2015). A path forward in the debate over health impacts of endocrine disrupting chemicals. Environ Health.

[16] Janesick A, Blumberg B (2012). Obesogens, stem cells and the developmental programming of obesity. Int J Androl.

[17] Heindel JJ: The Obesogen Hypothesis of Obesity: Overview and Human Evidence. In: Obesity Before Birth. Edited by Lustig RH. vol. 30. New York: Springer US; 2011;355–66.

[18] La Merrill M, Birnbaum LS (2011). Childhood obesity and environmental chemicals. Mt Sinai J Med.

[19] Mantovani A, Fucic A (2014). Puberty dysregulation and increased risk of disease in adult life: possible modes of action. Reprod Toxicol.

[20] Snedeker SM, Hay AG (2012). Do interactions between gut ecology and environmental chemicals contribute to obesity and diabetes?. Environ Health Perspect.

[21] Janesick A, Blumberg B (2011). Endocrine disrupting chemicals and the developmental programming of adipogenesis and obesity. Birth Defects Res C Embryo Today.

[22] Newbold RR (2011). Developmental exposure to endocrine-disrupting chemicals programs for reproductive tract alterations and obesity later in life. Am J Clin Nutrit.

[23] Mackay H, Patterson ZR, Khazall R, Patel S, Tsirlin D, Abizaid A (2013). Organizational effects of perinatal exposure to bisphenol-A and diethylstilbestrol on arcuate nucleus circuitry controlling food intake and energy expenditure in male and female CD-1 mice. Endocrinology.

[24] Newbold RR, Padilla-Banks E, Jefferson WN (2009). Environmental estrogens and obesity. Mol Cell Endocrinol.

[25] Angle BM, Do RP, Ponzi D, Stahlhut RW, Drury BE, Nagel SC, Welshons WV, Besch-Williford CL, Palanza P, Parmigiani S (2013). Metabolic disruption in male mice due to fetal exposure to low but not high doses of bisphenol A (BPA): Evidence for effects on body weight, food intake, adipocytes, leptin, adiponectin, insulin and glucose regulation. Reprod Toxicol.

[26] Somm E, Schwitzgebel VM, Toulotte A, Cederroth CR, Combescure C, Nef S, Aubert ML, Huppi PS (2009). Perinatal exposure to bisphenol a alters early adipogenesis in the rat. Environ Health Perspect.

[27] Skinner MK, Manikkam M, Tracey R, Guerrero-Bosagna C, Haque M, Nilsson EE (2013). Ancestral dichlorodiphenyltrichloroethane (DDT) exposure promotes epigenetic transgenerational inheritance of obesity. BMC Med.

[28] La Merrill M, Karey E, Moshier E, Lindtner C, La Frano MR, Newman JW, Buettner C (2014). Perinatal exposure of mice to the pesticide DDT impairs energy expenditure and metabolism in adult female offspring. PLoS One.

[29] Chamorro-Garcia R, Sahu M, Abbey RJ, Laude J, Pham N, Blumberg B (2013). Transgenerational inheritance of increased fat depot size, stem cell reprogramming, and hepatic steatosis elicited by prenatal exposure to the obesogen tributyltin in mice. Environ Health Perspect.

[30] Grun F, Watanabe H, Zamanian Z, Maeda L, Arima K, Cubacha R, Gardiner DM, Kanno J, Iguchi T, Blumberg B (2006). Endocrine-disrupting organotin compounds are potent inducers of adipogenesis in vertebrates. Mol Endocrinol.

[31] Hines EP, White SS, Stanko JP, Gibbs-Flournoy EA, Lau C, Fenton SE (2009). Phenotypic dichotomy following developmental exposure to perfluorooctanoic acid (PFOA) in female CD-1 mice: Low doses induce elevated serum leptin and insulin, and overweight in mid-life. Mol Cell Endocrinol.

[32] Hao C, Cheng X, Guo J, Xia H, Ma X (2013). Perinatal exposure to diethyl-hexyl-phthalate induces obesity in mice. Front Biosci (Elite Ed).

[33] Hao C, Cheng X, Xia H, Ma X (2012). The endocrine disruptor mono-(2-ethylhexyl) phthalate promotes adipocyte differentiation and induces obesity in mice. Biosci Rep.

[34] Manikkam M, Tracey R, Guerrero-Bosagna C, Skinner MK (2013). Plastics derived endocrine disruptors (BPA, DEHP and DBP) induce epigenetic transgenerational inheritance of obesity, reproductive disease and sperm epimutations. PLoS One.

[35] Stahlhut RW, van Wijgaarden E, Dye TD, Cook S, Swan SH (2007). Concentrations of urinary phthalate metabolites are associated with increased waist circumferece and insulin resistance in adult U.S. males. Environ Health Perspect.

[36] Hatch EE, Nelson JW, Qureshi MM, Weinberg J, Moore LL, Singer M, Webster TF (2008). Association of urinary phthalate metabolite concentrations with body mass index and waist circumference: a cross-sectional study of NHANES data, 1999–2002. Environ Health.

[37] Trasande L, Attina TM, Blustein J (2012). Association between urinary bisphenol A concentration and obesity prevalence in children and adolescents. JAMA.

[38] Trasande L, Spanier AJ, Sathyanarayana S, Attina TM, Blustein J (2013). Urinary Phthalates and Increased Insulin Resistance in Adolescents. Pediatrics.

[39] Lang IA, Galloway TS, Scarlett A, Henley WE, Depledge M, Wallace RB, Melzer D (2008). Association of urinary bisphenol A concentration with medical disorders and laboratory abnormalities in adults. JAMA.

[40] Tang-Peronard JL, Andersen HR, Jensen TK, Heitmann BL (2011). Endocrine-disrupting chemicals and obesity development in humans: A review. Obes Rev.

[41] Valvi D, Mendez MA, Garcia-Esteban R, Ballester F, Ibarluzea J, Goni F, Grimalt JO, Llop S, Marina LS, Vizcaino E (2014). Prenatal exposure to persistent organic pollutants and rapid weight gain and overweight in infancy. Obesity (Silver Spring).

[42] Skinner MK (2008). What is an epigenetic transgenerational phenotype? F3 or F2. Reprod Toxicol.

[43] Tracey R, Manikkam M, Guerrero-Bosagna C, Skinner MK (2013). Hydrocarbons (jet fuel JP-8) induce epigenetic transgenerational inheritance of obesity, reproductive disease and sperm epimutations. Reprod Toxicol.

[44] Wei J, Sun X, Chen Y, Li Y, Song L, Zhou Z, Xu B, Lin Y, Xu S (2014). Perinatal exposure to bisphenol A exacerbates nonalcoholic steatohepatitis-like phenotype in male rat offspring fed on a high-fat diet. J Endocrinol.

[45] Belcher SM, Chen Y, Yan S, Wang HS (2012). Rapid estrogen receptor-mediated mechanisms determine the sexually dimorphic sensitivity of ventricular myocytes to 17beta-estradiol and the environmental endocrine disruptor bisphenol A. Endocrinology.

[46] Nadal A: Obesity: Fat from plastics? Linking bisphenol A exposure and obesity. Nat Rev Endocrinol 2012, advance online publication. 10.1038/nrendo.2012.205 23147575

[47] Casals-Casas C, Desvergne B (2011). Endocrine disruptors: from endocrine to metabolic disruption. Annu Rev Physiol.

[48] Vandenberg LN, Colborn T, Hayes TB, Heindel JJ, Jacobs DR, Lee D-H, Shioda T, Soto AM, vom Saal FS, Welshons WV et al.: Hormones and Endocrine-Disrupting Chemicals: Low-Dose Effects and Nonmonotonic Dose Responses. Endocrine Reviews 2012;33(3):378–455. 10.1210/er.2011-1050 PMC3365860 22419778

[49] Bolton JL, Auten RL, Bilbo SD (2014). Prenatal air pollution exposure induces sexually dimorphic fetal programming of metabolic and neuroinflammatory outcomes in adult offspring. Brain Behav Immun.

