# Correction to: Parma consensus statement on metabolic disruptors

**DOI:** 10.1186/s12940-017-0343-0

**Published:** 2017-12-06

**Authors:** Jerrold J. Heindel, Frederick S. vom Saal, Bruce Blumberg, Patrizia Bovolin, Gemma Calamandrei, Graziano Ceresini, Barbara A. Cohn, Elena Fabbri, Laura Gioiosa, Christopher Kassotis, Juliette Legler, Michele La Merrill, Laura Rizzi, Ronit Machtinger, Alberto Mantovani, Michelle A. Mendez, Luisa Montanini, Laura Molteni, Susan C. Nagel, Stefano Parmigiani, Giancarlo Panzica, Silvia Paterlini, Valentina Pomatto, Jérôme Ruzzin, Giorgio Sartor, Thaddeus T. Schug, Maria E. Street, Alexander Suvorov, Riccardo Volpi, R. Thomas Zoeller, Paola Palanza

**Affiliations:** 1 Division of Extramural Research and Training, National Institute of Environmental Health Sciences, Research Triangle Park, NC USA; 2 Division of Biological Sciences, University of Missouri, Columbia, MO USA; 3 Department of Developmental and Cell Biology, University of California, Irvine, CA USA; 4 Department of Life Sciences and Systems Biology, University of Turin, Turin, Italy; 5 Department of Cell Biology and Neurosciences, Insituto Superiore di Sanita, Rome, Italy; 6 Department of Clinical and Experimental Medicine, University of Parma, Parma, Italy; 7 Public Health Institute, Berkeley, CA USA; 8 Interdepartment Center for Environmental Science Research, University of Bologna, Ravenna, Italy; 9 Department of Neuroscience, University of Parma, Parma, Italy; 10 Department of Toxicology and Environmental Health, VU University Amsterdam, Amsterdam, Netherlands; 11 Department of Environmental Toxicology, University of California, Davis, CA USA; 12 Department of Health Sciences, University of Milano-Bicocca, Monza, Italy; 13 Sheba Medical Center and Tel-Aviv University, Tel –Aviv, Israel; 14 Instituto Superiore di Sanita, Rome, Italy; 15 School of Public Health, University of North Carolina at Chapel Hill, Chapel Hill, NC USA; 16 Department of Pediatrics, University of Parma, Parma, Italy; 17 University of Milano-Bicocca, Monza, Italy; 18 Department of Obstetrics, Gynecology and Women’s Health, University of Missouri, Columbia, MO USA; 19 Faculty of Medicine, University of Parma, Parma, Italy; 20 Department of Neuroscience and Neuroscience Institute Cavalieri Ottolenghi (NICO), University of Turin, Turin, Italy; 21 Department of Biology, University of Bergen, Bergen, Norway; 22 Department of Pharmacy and Biotechnology, University of Bologna, Bologna, Italy; 23 Department of Pediatrics, University Hospital, Parma, Italy; 24 Department of Internal Medicine, University of Parma, Parma, Italy; 25 Department of Biology, University of Massachusetts, Amherst, MA USA

## Correction

After publication of the article [1], it has been brought to our attention that the thirteenth author of this article has had their name spelt incorrectly. In the original article the spelling “Laura Rizzir” was used. In fact the correct spelling should be “Laura Rizzi”.
